# Adulthood adiposity affects cardiac structure and function in later life

**DOI:** 10.1093/eurheartj/ehae403

**Published:** 2024-07-16

**Authors:** Lamia Al Saikhan, Nish Chaturvedi, Arjun K Ghosh, Rebecca Hardy, Alun Hughes

**Affiliations:** Department of Cardiac Technology, College of Applied Medial Sciences, Imam Abdulrahman Bin Faisal University, 2835 King Faisal Street, Dammam 34212, Saudi Arabia; MRC Unit for Lifelong Health and Ageing, Department of Population Science & Experimental Medicine, UCL Institute of Cardiovascular Science, University College London, Gower Street, London WC1E 6BT, UK; Barts Heart Centre, St Bartholomew’s Hospital, Barts Health NHS Trust, London, UK; Hatter Cardiovascular Institute, University College London Hospital NHS Foundation Trust, London, UK; School of Sport, Exercise, and Health Sciences, Loughborough University, Loughborough, UK; MRC Unit for Lifelong Health and Ageing, Department of Population Science & Experimental Medicine, UCL Institute of Cardiovascular Science, University College London, Gower Street, London WC1E 6BT, UK

**Keywords:** Obesity, Adiposity gain, Cardiac structure, Cardiac function, LV mass, LV diastolic function, Adult life course

## Abstract

**Background and Aims:**

Excess adiposity is associated with poorer cardiac function and adverse left ventricular (LV) remodelling. However, its importance over the adult life course on future cardiac structure and systolic and diastolic function is unknown.

**Methods:**

A total of 1690 participants in the National Survey of Health and Development birth cohort underwent repeated adiposity [body mass index (BMI)/waist-to-hip ratio (WHR)] measurements over adulthood and investigation, including echocardiography at age 60–64 years. The relationship between LV structure [LV mass (LVM), relative wall thickness, and LV internal diameter in diastole (LVIDd)] and function (diastolic: E/e*ʹ*, e*ʹ*, and left atrial volume indexed to body surface area; systolic: ejection fraction, S*ʹ*, and myocardial contraction fraction) was investigated using multivariable linear regression models.

**Results:**

Increased BMI from age 20 years onwards was associated with greater LVM and LVIDd independent of confounders. Associations remained independent of current BMI for LVIDd and at age 26, 43, and 53 years for LVM. Increased BMI from 43 years onwards was associated with greater relative wall thickness, but not when BMI at age 60–64 years was accounted for. Increased BMI at age 26, 36, and 53 years and at 20 years onwards was associated with lower ejection fraction and myocardial contraction fraction, respectively, but not independently of BMI at 60–64 years. Higher BMI from 20 years onwards was associated with poorer diastolic function independent of confounders. Associations between BMI and left atrial volume indexed to body surface area persisted from 26 years onwards after adjustment for BMI at 60–64 years. Similar relationships were observed for WHR from age 43 years onwards.

**Conclusions:**

Higher adiposity (BMI/WHR) over adulthood is associated with evidence of adverse cardiac structure and function. Some of these associations are independent of adiposity in later life.

## Structured Graphical Abstract

**Structured Graphical Abstract:**
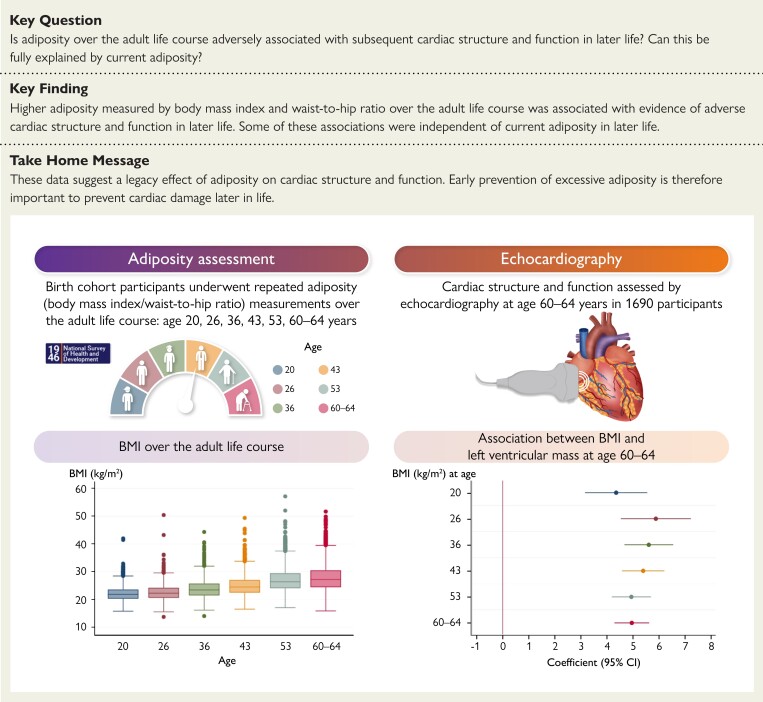
Excess adiposity over the adult life course and its associations with cardiac structure and function in later life. BMI, body mass index; CI, confidence interval.

**See the editorial comment for this article ‘Lifelong impact of adiposity on cardiac structure and function: an alarming signal’, by L. Roever *et al*., https://doi.org/10.1093/eurheartj/ehae443.**

## Introduction

Worldwide obesity has nearly tripled since 1975 with 39% of adults aged ≥18 years being classified as overweight in 2016 and 13% obese according to the World Health Organization.^1^ Obesity and overweight are associated with an increased risk of cardiovascular disease (CVD), including heart failure, and mortality.^2,3^

In cross-sectional studies, elevated body mass index (BMI) is associated with poorer cardiac function (i.e. poorer diastolic and systolic function) and adverse left ventricular (LV) remodelling [i.e. elevated LV mass (LVM) and increased LV cavity size] albeit with inconsistent relationships across studies.^4–6^ However, evidence on the relationship between excess adiposity over the adult life course and future cardiac structure and function, and its reversibility is limited. This is important because understanding the temporal relationship between adiposity gain and cardiac measures provides insight into effective preventative strategies.

This study, therefore, aimed to investigate the relationship of adiposity, measured by BMI/waist-to-hip ratio (WHR), over the adult life course with cardiac structure and function, measured by echocardiography, at age 60–64 years based on longitudinal data derived from the Medical Research Council National Survey of Health and Development (MRC NSHD) birth cohort. We hypothesized that antecedent adiposity prior to age 60–64 would be adversely associated with subsequent cardiac structure and function in later life, and that this would not be completely explained by current adiposity.

## Methods

### Study population

The MRC NSHD is a birth cohort study consisting of 5362 individuals born in March 1946 in England, Scotland, and Wales.^7^ The design of the study has been described previously.^7,8^ In brief, the whole sample has been followed up over 20 times since birth with adult BMI ascertained at 20, 26, 36, 43, and 53 years. At age 60–64 years (2006–10), 2856 eligible study members were invited to participate, 2229 (78%) responded, and of these, 1690 (59.1%) attended clinic, and 539 (18.9%) had a home visit (see Supplementary data online, *Figure S1*). Clinic attenders underwent a comprehensive examination that included health, lifestyle, and sociodemographic questionnaires, height and weight measurements, and detailed cardiovascular assessments.^7,8^ Data from individuals with echocardiographic measurements were used in this analysis. The study received regional Ethics Committee approval (MREC98/2/121, 08/MRE00/12, and 07/H1008/245) and was performed in accordance with the principles of the Helsinki Declaration. All participants provided written informed consent for each component of data collection.

### Clinical examination

At the clinic visit, anthropometrics including height and weight were measured in light indoor clothing without shoes and BMI was calculated. Waist and hip circumferences were measured to the nearest millimetres using identical protocol from age 43 years onwards, and WHR was derived. Waist measurements were taken at the midpoint between the costal margin and the iliac crest, and hip at the level of the greater trochanter on skin or under light clothing. Two sitting blood pressure measurements from the upper right arm were recorded in clinic using an appropriately sized cuff after resting for 5 min, and the average of the two readings was used. Socioeconomic position was defined as the highest household occupational social class at 53 years [using Registrar General’s Social Class (RGSC)].^9^ Education was defined as the highest educational level achieved by 26 years classified using the Burnham scale as follows: (i) no qualifications, (ii) sub-GCE or sub-Burnham C, (iii) GCE O level or Burnham C, (iv) GCE A level or Burnham B, and (v) degree or higher. The presence of Type 2 diabetes mellitus at 60–64 years was defined based on self-report or clinic blood tests.

### Echocardiographic imaging and analysis

Of the 1690 participants who attended clinic, 1653 participants underwent echocardiography using GE Vivid I machines (General Electric, Fairfield, Connecticut, USA) performed by trained experienced sonographers according to a strict protocol. The imaging protocol included the following echocardiographic views: parasternal long axis and short axis, apical five-, four-, three-, two-chamber, and aortic views in addition to conventional and tissue Doppler in the four-chamber view. Images were optimized if required using second harmonic imaging. Image analysis was performed by three experienced echocardiographers blinded to patient identity using GE EchoPAC Software (GE Connecticut, USA) in a single core laboratory.

Left ventricular wall thickness and cavity dimensions from 2D-guided M-mode were measured from parasternal long-axis view from which LV relative wall thickness (RWT) was calculated.^10^ Left ventricular mass was also calculated following the American Society of Echocardiography (ASE) recommendations.^10^ Indexation to body size was achieved by adjusting LVM for height^1.7^,^11^ since indexing to body surface area is known to bias results when there is obesity.^10^ Left ventricular volumes and LV ejection fraction (EF) was calculated using the modified biplane Simpson’s rule.^10^ Diastolic function was evaluated in accordance with the ASE recommendations and included the following markers: mitral inflow diastolic filling velocities [early (E-wave), late (A-wave), and E*/*A ratio] and tissue Doppler analysis averaged of the lateral and septal mitral annular velocities (eʹ, aʹ, and sʹ) from which E*/*e*ʹ* ratio was calculated.^10^ Maximum left atrial volume [indexed (LAVi)] to BMI was calculated by the biplane method of discs at end-systole as a marker of chronically elevated LV filling pressures, and indexation to height and height^2^ was also performed in sensitivity analyses.^10,12,13^ Myocardial contraction fraction (MCF), a measure of LV myocardial shortening, was calculated as the ratio of LV stroke volume to LV myocardial volume.

Quality checks were performed throughout the study including blind duplicate reading reproducibility studies (*n* = 40; in similar numbers of men and women and with varying image qualities) to establish inter- and intrareader variability. The reproducibility was excellent with intraclass correlation coefficients >0.8 for all measurements.

### Statistical analysis

Statistical analyses were performed using STATA 15.1 (StataCorp LLC, College Station, TX, USA). Continuous variables are presented as mean ± standard deviation or median (interquartile range) as appropriate, and categorical variables are presented as counts (percentages). The relationship between BMI at each of the six ages when it was measured and cardiac structural and functional measures at 60–64 years was investigated using multivariable linear regression models, and regression coefficients with 95% confidence intervals (CIs) are reported. Models were adjusted for prespecified covariables as follows: Model 1: adjusted for age and sex [plus height for LV internal diameter in diastole (LVIDd); and height^1.7^ for LVM]; Model 2: Model 1 plus occupational social class and education; and Model 3: Model 2 plus current BMI. The analysis was repeated replacing BMI, as a measure of adiposity, with WHR, which was available at NSHD only from age 43 onwards. Model 3 aimed to establish whether antecedent adiposity (BMI/WHR) was predictive of cardiac structure and function independent of current adiposity. A sensitivity analysis was performed omitting participants with Type 2 diabetes mellitus (T2DM). Sex-stratified analyses were performed as a sensitivity analysis, and interaction *P* < .05 was taken as indicative of sex modification of associations. Sex-stratified models are reported in the supplement for completeness, although results did not differ markedly from pooled models. Missing values were rare and were dealt with by listwise deletion, which is valid when missingness is rare and data are missing completely at random. Inference was made on the basis of two-tailed *P*-values, CIs, and effect size.

## Results

### Population characteristics

**Figure 1:**
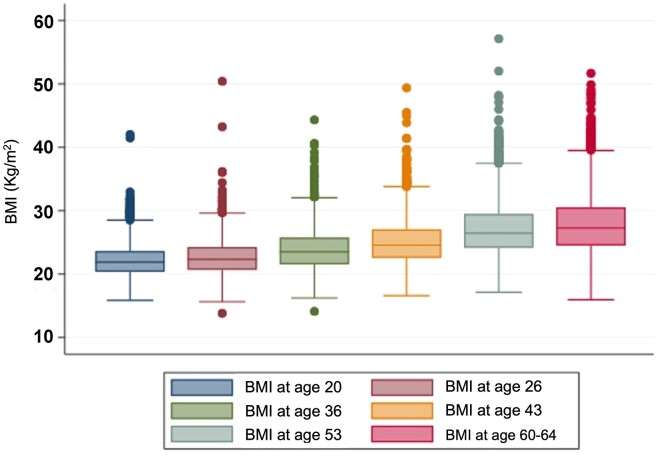
The mean body mass index at each of the six age periods at which it was measured, demonstrating a steady increase in median body mass index with increasing age

Sample characteristics are summarized in *Table 1*. Mean age at outcome measurement was 63.2 ± 1.1 years, and 48.3% were male. *Figure 1* illustrates the increase in mean BMI with age over the adult life course.

**Table 1:** Echocardiographic and cardiac risk factor characteristics of the study sample

Variable (at age 60–64 years unless stated otherwise)	*n* (%)	Mean (SD)
Age at echo, years	1638	63.2 (1.1)
Male	791 (48.3)	
Socioeconomic status	1639	
Professional	137 (8.4)	
Intermediate	726 (44.3)	
Skilled (non-manual)	385 (23.5)	
Skilled (manual)	215 (13.1)	
Partly skilled	130 (7.9)	
Unskilled	37 (2.3)	
Education	1639	
None attempted	391 (23.9)	
Vocational course, proficiency only	44 (2.7)	
Sub-GCE or sub-Burnham C	68 (4.2)	
GCE ‘O’ level or Burnham C	340 (20.7)	
GCE ‘A’ level or Burnham B	269 (16.4)	
Burnham A2	228 (13.9)	
1st degree or graduate equivalent	198 (12.1)	
Higher degree, master’s or doctorate	18 (1.1)	
Unknown	52 (3.2)	
No data	31 (1.9)	
Smoking status	1639	
Current smoker	144 (8.8)	
Ex-smoker	620 (37.8)	
Never smoked	739 (45.1)	
Unknown	136 (8.3)	
Interventricular septum, cm	1479	1.1 (0.25)
Posterior wall thickness, cm	1473	1.0 (0.18)
Relative wall thickness	1473	0.42 (0.1)
LV mass, g	1473	182.1 (62.1)
LV mass/height^1.7^, g/m^1.7^	1473	74.7 (23.6)
LV internal diameter at diastole, cm	1494	4.8 (0.6)
LV internal diameter at systole, cm	1498	2.9 (0.5)
E-wave, cm/s	1576	0.67 (0.15)
A-wave, cm/s	1578	0.70 (0.17)
E/A ratio	1576	1.0 (0.27)
e*ʹ*, cm/s	1600	8.9 (1.9)
E/e*ʹ* ratio	1558	7.9 (2.2)
LA volume index, mL/m^2^	1404	23.6 (8.8)
LV ejection fraction, %	1447	64.3 (7.8)
S*ʹ*, cm/s	1505	7.8 (1.6)
Myocardial contraction fraction, %	1470	45.7 (12.8)
BMI, kg/m^2^	1639	27.6 (4.6)
BMI at age 53, kg/m^2^	1555	26.9 (4.4)
BMI at age 43, kg/m^2^	1544	24.9 (3.7)
BMI at age 36, kg/m^2^	1486	23.7 (3.2)
BMI at age 26, kg/m^2^	1445	22.5 (2.8)
BMI at age 20, kg/m^2^	1352	22.0 (2.6)
Waist-to-hip ratio	1634	0.91 (0.1)
Waist-to-hip ratio at age 53	1558	0.86 (0.1)
Waist-to-hip ratio at age 43	1425	0.83 (0.1)
Systolic blood pressure, mmHg	1634	136.6 (17.8)
Diastolic blood pressure, mmHg	1635	77.9 (9.6)
Heart rate, b.p.m.	1637	68.5 (11.2)
T2DM	105 (6.4)	
HbA1c, %	1534	5.8 (0.6)
Glucose, mmol/L	1525	3.9 (1.2)
Creatinine, mmol/L	1525	0.06 (0.02)
Cystatin C, mg/L	1570	0.81 (0.13)

A, late mitral inflow diastolic filling velocity; BMI, body mass index; e*ʹ*, early diastolic mitral annular velocity; E, early mitral inflow diastolic filling velocity; HbA1c, glycosylated haemoglobin; LA, left atrium; LV, left ventricle; S*ʹ*, systolic mitral annular velocity; T2DM, Type 2 diabetes mellitus.

### Associations between left ventricular structural measures and adiposity at various ages

**Figure 2:**
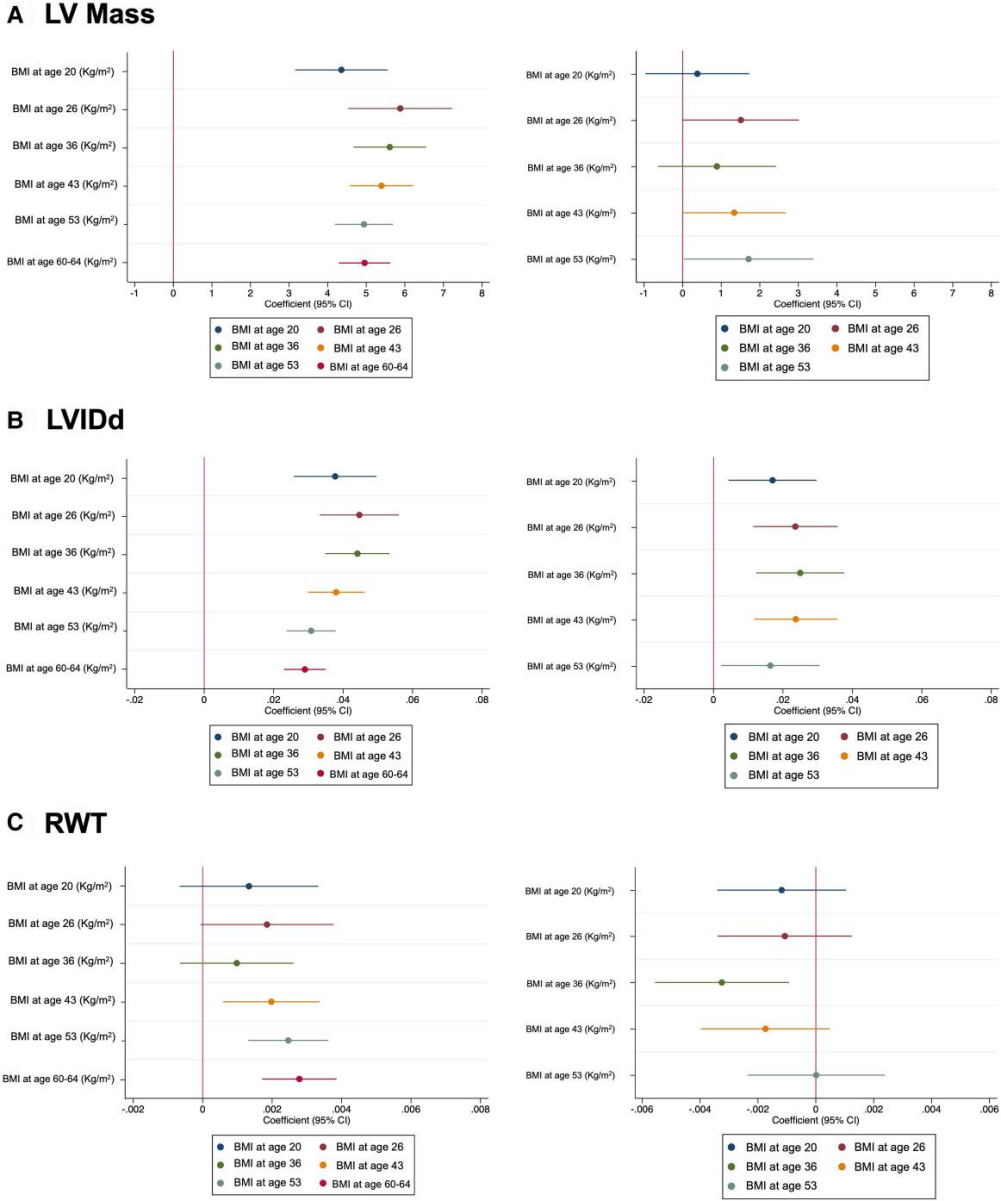
Association between body mass index at different ages and left ventricular structure measured at age 60–64 years. A) Left ventricular (LV) mass, B) left ventricular internal diameter in diastole (LVIDd) and C) relative wall thickness (RWT). Left column: Model 2 adjusted for age and sex (plus height for left ventricular internal diameter in diastole and height^1.7^ for left ventricular mass), socioeconomic status/social class, and education. Right column: Model 3 adjusted for Model 2 plus current body mass index

The associations of BMI at different ages with LVM, LVIDd, and RWT are presented in *Figure 2* (and Supplementary data online, *Figure S2* and *Table S1*). Increased BMI from the age of 20 years onwards was associated with greater LVM in confounder-adjusted models (Model 1, Model 2). When BMI at age 60–64 years was included in models, the strength of association of BMI at prior ages was substantially attenuated for LVM, although there was still evidence of associations at age 26, 43, and 53 years independent of current BMI.

Increased BMI from the age of 20 years onwards was associated with greater LVIDd in confounder-adjusted models (Model 1, Model 2). When BMI at age 60–64 years was included in models, the effect of BMI at prior ages was attenuated slightly, but the associations remained independent of current BMI.

Higher BMI from age 43 years onwards was associated with greater RWT in confounder-adjusted models (Model 1, Model 2). At younger ages, associations were in the same direction but did not convincingly differ from zero. When BMI at age 60–64 years was included in models, the associations were attenuated to the null and in one case (age 36 years) reversed.

Similar relationships were observed for WHR from age 43 onwards (see Supplementary data online, *Table S2*), with the exception of LVIDd at age 43, and all associations (i.e. LVM, LVIDd, and RWT) were independent of current WHR.

### Associations between left ventricular functional measures and adiposity at various ages

#### Systolic function

**Figure 3:**
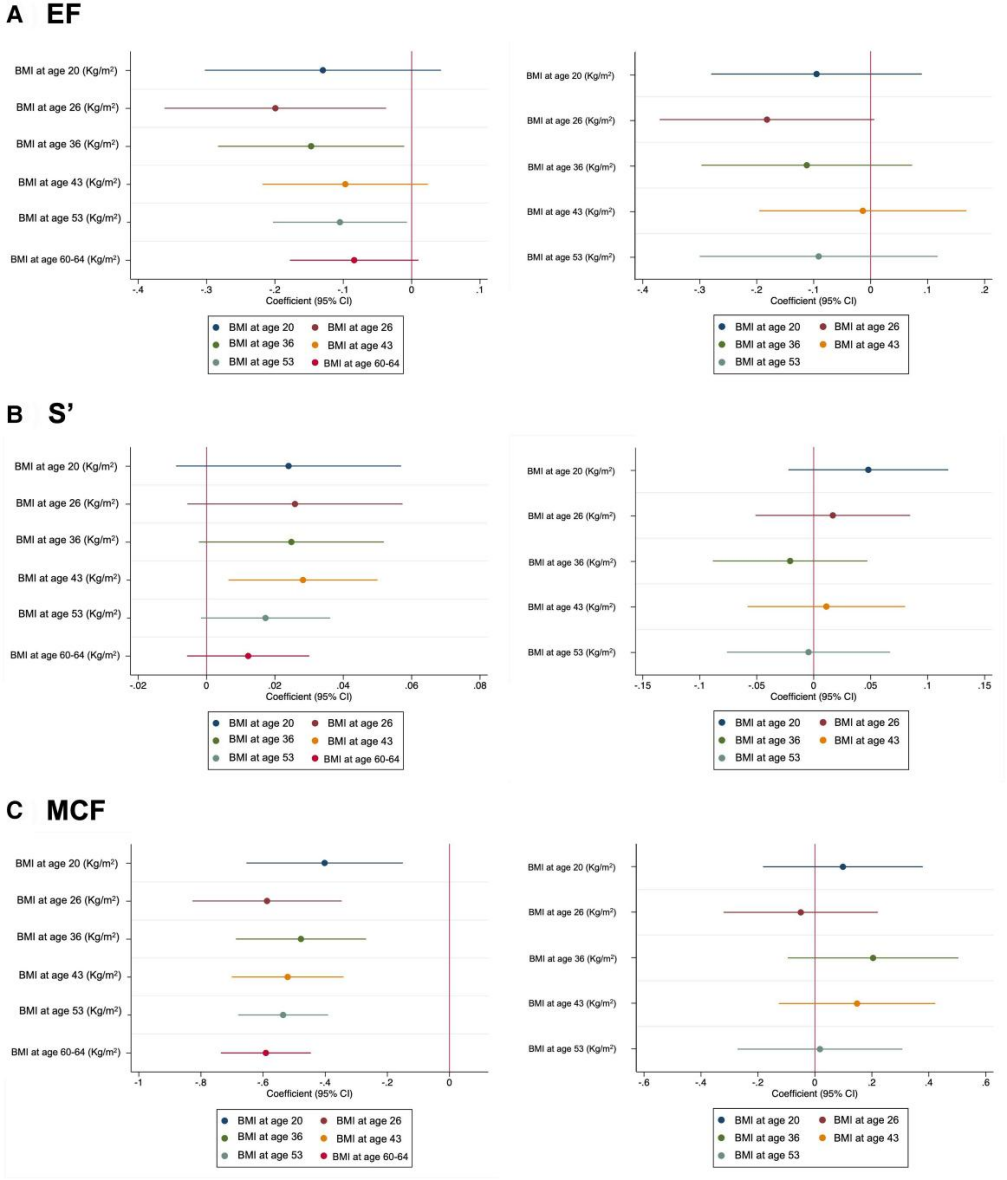
Association between body mass index at different ages and left ventricular systolic function measured at age 60–64 years. A) Ejection fraction (EF), B) systolic mitral annular velocity (S') and C) myocardial contraction fraction (MCF). Left column: Model 2 adjusted for age and sex, socioeconomic status/social class, and education. Right column: Model 3 adjusted for Model 2 plus current body mass index

The associations of BMI at different ages with EF, S*ʹ*, and MCF are presented in *Figure 3* (and Supplementary data online, *Figure S3* and *Table S3*). Higher BMI at age 26, 36, and 53 years was associated with lower EF, i.e. worse systolic function in confounder-adjusted models (Model 1, Model 2). When BMI at age 60–64 years was included in models, the associations were attenuated to null.

Most associations between higher BMI and S*ʹ* were unconvincing, although there was some evidence that higher BMI at age 43 years was associated with higher S*ʹ*. After adjusting for current BMI, there was no evidence of associations with S*ʹ*.

Higher BMI from age 20 years onwards was associated with a lower MCF, i.e. worse systolic function in confounder-adjusted models (Model 1, Model 2). When BMI at age 60–64 years was included in models, all associations were abolished.

Similar associations were observed for WHR from age 43 onwards (see Supplementary data online, *Table S4*), although associations between higher WHR from age 43 onwards and lower MCF (i.e. worse systolic function) were independent of WHR at age 60–64 years.

#### Diastolic function

**Figure 4:**
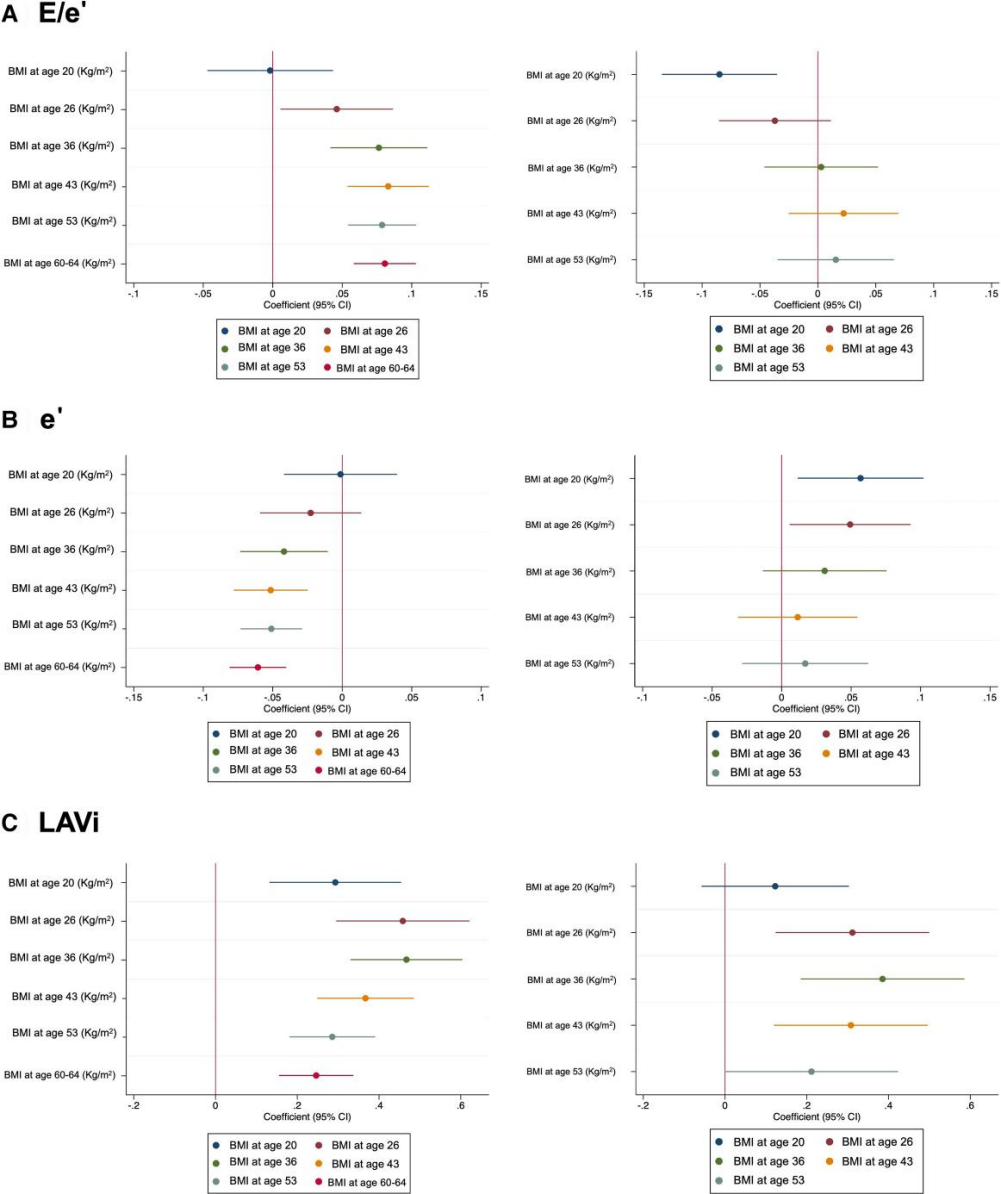
Association between body mass index at different ages and left ventricular diastolic function measured at age 60–64 years. A) Ratio of early mitral inflow diastolic filling velocity to early diastolic mitral annular velocity (E/e'), B) early diastolic mitral annular velocity (e') and C) left atrial volume index (LAVi). Left column: Model 2 adjusted for age and sex, socioeconomic status/social class, and education. Right column: Model 3 adjusted for model 2 plus current body mass index

The associations of BMI at different ages with E/e*ʹ*, e*ʹ*, and LAVi are presented in *Figure 4* (and Supplementary data online, *Figure S4* and *Table S5*). Higher BMI from the age of 26 years onwards was associated with higher E/e*ʹ*, i.e. worse diastolic function in confounder-adjusted models (Model 1, Model 2). When BMI at 60–64 years was included in the models, no convincing associations were evident at ages 26–53 years and the association became negative at age 20 years, i.e. higher BMI at age 20 years was associated with lower E/e*ʹ*, i.e. better diastolic function after accounting for current BMI.

Increased BMI from the age of 36 years onwards was associated with lower e*ʹ*, i.e. worse diastolic function in confounder-adjusted models. On including BMI at age 60–64 (Model 3), the associations at age 36 years and above were reversed at age 20 and 26 years.

Increased BMI from the age of 20 years onwards was associated with greater LAVi in confounder-adjusted models (Model 1, Model 2). On including BMI at age 60–64, associations were moderately attenuated, although there was evidence of independent associations from age 26 years onwards.

Similar relationships were observed for WHR from age 43 onwards (see Supplementary data online, *Table S6* ) with associations between WHR and LAVi being independent of current WHR. Results stratified by sex are shown in Supplementary data online, (Figures S5–S7). Exclusion of people with T2DM had little impact on associations (Supplementary data online, Tables S7–S9).

## Discussion

In a birth cohort of men and women aged 60–64 years, excess adiposity measured as higher BMI or WHR from early or mid-adulthood was associated with adverse cardiac structure and function. For LVM, RWT, and LVIDd, associations with adiposity after age 43 years were independent of adiposity at age 60–64 years, consistent with an enduring influence of adiposity earlier in life. Higher adiposity (BMI/WHR) from early adulthood onwards was also associated with evidence of poorer systolic and diastolic function in older age. These associations were mostly explained by adiposity at age 60–64 years, but in the case of LAVi and MCF, there was evidence of adverse associations with antecedent adiposity independent of current adiposity (*Structured Graphical Abstract*).

Our study adds to existing knowledge. Previous cross-sectional studies have shown that elevated BMI is associated with adverse LV remodelling (i.e. elevated LVM and increased LV cavity size), poorer systolic and diastolic function, and increased risk of heart failure.^4–6,14–16^ Based on Mendelian randomization studies, it is likely that at least some of these relationships are causal.^17,18^ A few previous studies have reported a relationship between high BMI in childhood and adverse cardiac structure in later life,^19–21^ and in adulthood, increasing BMI over a decade of adult life has been reported to be associated with higher LVM.^22^ However, we are unaware of previous studies that have examined the association between BMI (and/or WHR) measured repeatedly across adulthood with cardiac measures in older life and that also account for the influence of adiposity in older life.

Our findings show that excess adiposity over the life course is associated with adverse cardiac structure and function that is only partly attributable to tracking of BMI (and/or WHR where applicable). This suggests a lasting impact of being overweight/obese early in adulthood on the heart. There was no compelling evidence of a sensitive period during adulthood when associations between BMI (and/or WHR) and later life cardiac outcomes were markedly stronger than at other ages, although most associations with BMI in earlier adulthood (below 36 years) were weaker than those subsequently. It is possible that interpreting BMI as a measure of adiposity is less secure at these younger ages since muscle mass will also contribute to BMI, especially in a cohort where BMI was largely in the normal range at younger ages.

### Limitations

The MRC NSHD was a representative sample of native-born adults living in England, Scotland, and Wales at the time of data collection.^7,23^ However, our findings cannot be generalized beyond the White British population of similar age. The repeated measurements of adiposity in our study, the longest running birth cohort in Britain, allowed us a rare opportunity to perform a longitudinal analysis across adult life investigating the associations of adiposity and changes in adiposity with cardiac structure and function using echocardiography in a large sample of people. Heights and weights recorded at ages 20 and 26 years were based on self-report and thus could be subject to reporting bias. Unmeasured or residual confounders that influence BMI or WHR and cardiac structure and function may have biased the associations observed. Probably the most important of these is likely to be dietary calorie intake; this was not accounted for in our models. Our analyses examined multiple associations, which increase the possibility of ‘false discovery’.

### Future directions

Our data suggest lasting effects of adiposity on cardiac structure and function and reinforce calls for system approaches to the obesity syndemic.^24^ Early prevention of excessive adiposity gain may be important to prevent future cardiac target organ damage in later life. This possibility should be explored in future studies, including in more diverse populations.

## Conclusions

Higher adiposity (measured by BMI/WHR) over adult life is associated with evidence of adverse cardiac structure and function. Some of these associations are independent of adiposity in later life.

## Supplementary Material

ehae403_Supplementary_Data

## Declarations

### Data Availability

Data used in this publication are available to bona fide researchers on request to the National Survey of Health and Development Data Sharing Committee via a standard application procedure. Further details can be found at http://www.nshd.mrc.ac.uk/data, https://doi.org/10.5522/NSHD/Q101, https://doi.org/10.5522/NSHD/Q102, and https://doi.org/10.5522/NSHD/Q103.

### Pre-registered Clinical Trial Number

None supplied.
