# Supplementary material for: Adulthood adiposity affects cardiac structure and function in later life
Source: Eur Heart J. 2024 Jul 16;45(33):3060–8. doi: 10.1093/eurheartj/ehae403 (PMC11365607; doi:10.1093/eurheartj/ehae403)
Supplement: ehae403_Supplementary_Data [file ehae403_supplementary_data.docx]

**Supplements**


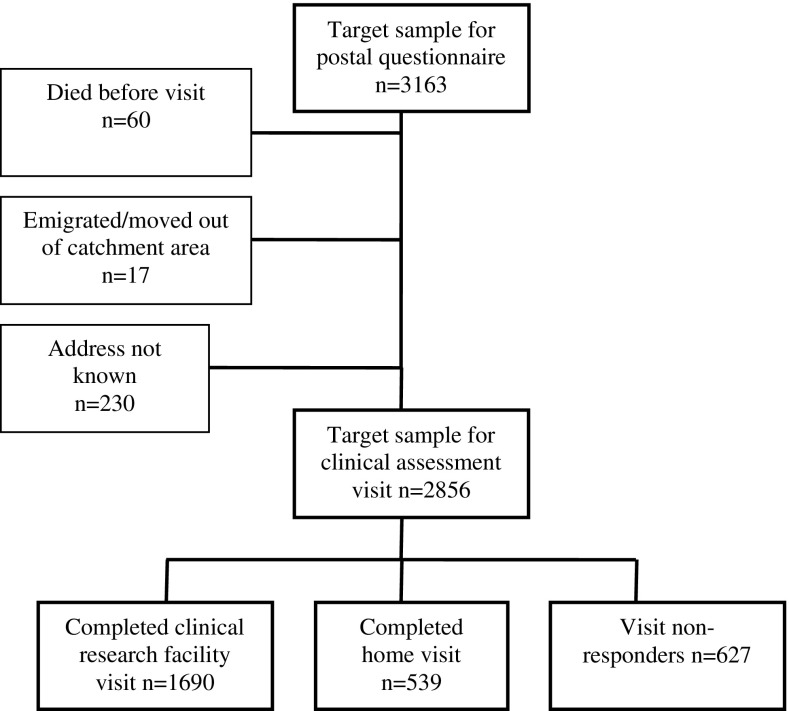


**Figure-S1. Flow chart summarising response status at 60-64 years.**

**Figure-S2. Association between BMI at different ages and LV structure measured at age 60-64y**. Model-1 adjusted for age and sex (plus height for LVIDd; and height^1.7^ for LV mass).

**Figure-S3. Association between BMI at different ages and measures of LV systolic function measured at age 60-64y**. Model-1 adjusted for age and sex.

**Figure-S4. Association between BMI at different ages and measures of LV diastolic function measured at age 60-64y**. Model-1 adjusted for age and sex. LAVi to BSA.

| **Table S1. Association between BMI at different ages and LV structure measured at age 60-64y** | | | | | | |
| --- | --- | --- | --- | --- | --- | --- |
| **Age (y)** | **Model-1** | | **Model-2** | | **Model-3** | |
|  | **Coefficient (95% CI)** | **P value** | **Coefficient (95% CI)** | **P value** | **Coefficient (95% CI)** | **P value** |
| **LV mass, g** | | | | | | |
| **20y (n=1222)** | 4.4973 (3.3062, 5.6883) | <0.0001 | 4.3558 (3.1589, 5.5526) | <0.0001 | 0.3815 (-0.9638, 1.7268) | 0.578 |
| **26y (n=1303)** | 6.0603 (4.7075, 7.4131) | <0.0001 | 5.8806 (4.5361, 7.2250) | <0.0001 | 1.5110 (0.0095, 3.0125) | 0.049 |
| **36y (n=1336)** | 5.7831 (4.8590, 6.7073) | <0.0001 | 5.6092 (4.6720, 6.5464) | <0.0001 | 0.8882 (-0.6412, 2.4176) | 0.255 |
| **43y (n=1395)** | 5.5417 (4.7249, 6.3585) | <0.0001 | 5.3933 (4.5802, 6.2065) | <0.0001 | 1.3372 (0.0042, 2.6702) | 0.049 |
| **53y (n=1406)** | 5.0218 (4.2639, 5.7797) | <0.0001 | 4.9405 (4.1907, 5.6902) | <0.0001 | 1.7090 (0.0293, 3.3887) | 0.046 |
| **60-64y (n=1475)** | 5.0622 (4.3937, 5.7308) | <0.0001 | 4.9569 (4.2906, 5.6232) | <0.0001 |  |  |
| **LVIDd, cm** | | | | | | |
| **20y (n=1239)** | 0.0384 (0.0264, 0.0503) | <0.0001 | 0.0377 (0.0258, 0.0496) | <0.0001 | 0.0170 (0.0043, 0.0296) | 0.009 |
| **26y (n=1322)** | 0.0457 (0.0339, 0.0575) | <0.0001 | 0.0446 (0.0333, 0.0560) | <0.0001 | 0.0235 (0.0114, 0.0357) | <0.0001 |
| **36y (n=1355)** | 0.0460 (0.0368, 0.0551) | <0.0001 | 0.0444 (0.0348, 0.0533) | <0.0001 | 0.0249 (0.0122, 0.0376) | <0.0001 |
| **43y (n=1416)** | 0.0393 (0.0311, 0.0475) | <0.0001 | 0.0380 (0.0298, 0.0461) | <0.0001 | 0.0237 (0.0117, 0.0356) | <0.0001 |
| **53y (n=1426)** | 0.0310 (0.0238, 0.0381) | <0.0001 | 0.0308 (0.0237, 0.0378) | <0.0001 | 0.0163 (0.002, 0.0305) | 0.023 |
| **60-64y (n=1496)** | 0.0301 (0.0242, 0.0360) | <0.0001 | 0.0289 (0.0230, 0.0349) | <0.0001 |  |  |
| **RWT** | | | | | | |
| **20y (n=1222)** | 0.0014 (-0.0005, 0.0033) | 0.165 | 0.0013 (-0.0006, 0.0033) | 0.191 | -0.0011 (-0.0034, 0.0010) | 0.298 |
| **26y (n=1303)** | 0.0019 (0.0000, 0.0038) | 0.040 | 0.0018 (-0.0000, 0.0037) | 0.058 | -0.0010 (-0.0033, 0.0012) | 0.366 |
| **36y (n=1336)** | 0.0009 (-0.0006, 0.0025) | 0.241 | 0.0009 (-0.0006, 0.0026) | 0.240 | -0.0032 (-0.0055, -0.0009) | 0.006 |
| **43y (n=1395)** | 0.0019 (0.0005, 0.0033) | 0.005 | 0.0019 (0.0005, 0.0033) | 0.005 | -0.0017 (-0.0039, 0.0004) | 0.126 |
| **53y (n=1406)** | 0.0025 (0.0013, 0.0036) | <0.0001 | 0.0024 (0.0013, 0.0036) | <0.0001 | 0.0000 (-0.0023, 0.0023) | 0.992 |
| **60-64y (n=1475)** | 0.0027 (0.0016, 0.0037) | <0.0001 | 0.0027 (0.0017, 0.0038) | <0.0001 |  |  |

**Model-1: adjusted for age and sex (plus height for LVIDd; and height^1.7^ for LV mass); model-2: adjusted for model-1 plus socioeconomic status/social class and education; and model-3: adjusted for model-2 plus current BMI.**

| **Table S2. Association between WHR at different ages and LV structure measured at age 60-64y** | | | | | | |
| --- | --- | --- | --- | --- | --- | --- |
| **Age (y)** | **Model-1** | | **Model-2** | | **Model-3** | |
|  | **Coefficient (95% CI)** | **P value** | **Coefficient (95% CI)** | **P value** | **Coefficient (95% CI)** | **P value** |
| **LV mass, g** | | | | | | |
| **43y (n=1287)** | 212.3 (159.3, 265.3) | <0.0001 | 201.6 (148.6, 254.7) | <0.0001 | 125.8 (68.7, 182.8) | <0.0001 |
| **53y (n=1407)** | 250.0 (198.4, 301.5) | <0.0001 | 244.6 (193.0, 296.1) | <0.0001 | 182.3 (122.9, 241.6) | <0.0001 |
| **60-64y (n=1471)** | 212.3 (165.8, 258.9) | <0.0001 | 202.5 (156.2, 248.8) | <0.0001 |  |  |
| **LVIDd, cm** | | | | | | |
| **43y (n=1306)** | 0.8825 (0.3189, 1.446) | 0.002 | 0.7370 (0.1732, 1.300) | 0.010 | 0.2342 (-0.3841, 0.8525) | 0.458 |
| **53y (n=1427)** | 1.434 (0.9798, 1.889) | <0.0001 | 1.341 (0.8843, 1.799) | <0.0001 | 1.063 (0.4980, 1.629) | <0.0001 |
| **60-64y (n=1492)** | 1.144 (0.7409, 1.548) | <0.0001 | 1.010 (0.6014, 1.419) | <0.0001 |  |  |
| **RWT** | | | | | | |
| **43y (n=1287)** | 0.1929 (0.1037, 0.2820) | <0.0001 | 0.2008 (0.1099, 0.2917) | <0.0001 | 0.1749 (0.0738, 0.2760) | 0.001 |
| **53y (n=1407)** | 0.1458 (0.0619, 0.2297) | 0.001 | 0.1511 (0.0661, 0.2361) | <0.0001 | 0.1063 (0.0023, 0.2102) | 0.045 |
| **60-64y (n=1471)** | 0.1228 (0.0458, 0.1998) | 0.002 | 0.1298 (0.0515, 0.2081) | 0.001 |  |  |

**Model-1: adjusted for age and sex (plus height for LVIDd; and height^1.7^ for LV mass); model-2: adjusted for model-1 plus socioeconomic status/social class and education; and model-3: adjusted for model-2 plus current WHR.**

| **Table S3. Association between BMI at different ages and LV systolic function measured at age 60-64y** | | | | | | |
| --- | --- | --- | --- | --- | --- | --- |
| **Age (y)** | **Model-1** | | **Model-2** | | **Model-3** | |
|  | **Coefficient (95% CI)** | **P value** | **Coefficient (95% CI)** | **P value** | **Coefficient (95% CI)** | **P value** |
| **EF, %** | | | | | | |
| **20y (n=1195)** | -0.1126 (-0.2836, 0.0584) | 0.197 | -0.1298 (-0.3025, 0.0428) | 0.141 | -0.0949 (-0.2798, 0.0899) | 0.314 |
| **26y (n=1279)** | -0.1747 (-0.3313, -0.0180) | 0.029 | -0.1993 (-0.3613, -0.0373) | 0.016 | -0.1820 (-0.3703, 0.0062) | 0.058 |
| **36y (n=1312)** | -0.1329 (-0.2649, -0.0009) | 0.048 | -0.1470 (-0.2832, -0.0108) | 0.034 | -0.1121 (-0.2971, 0.0727) | 0.234 |
| **43y (n=1370)** | -0.0870 (-0.2031, 0.0289) | 0.141 | -0.0970 (-0.2178, 0.0237) | 0.115 | -0.0137 (-0.1954, 0.1679) | 0.882 |
| **53y (n=1373)** | -0.1017 (-0.1966, -0.0068) | 0.036 | -0.1048 (-0.2028, -0.0069) | 0.036 | -0.0912 (-0.3000, 0.1176) | 0.392 |
| **60-64y (n=1449)** | -0.0787 (-0.1700, 0.0125) | 0.091 | -0.0804 (-0.1780, -0.0100) | 0.080 |  |  |
| **S’, cm/s** | | | | | | |
| **20 (n=1242)** | 0.0274 (-0.0056, 0.0604) | 0.104 | 0.0239 (-0.0089, 0.0569) | 0.153 | 0.0134 (-0.0232, 0.0501) | 0.472 |
| **26 (n=1328)** | 0.0251 (-0.0060, 0.0563) | 0.114 | 0.0258 (-0.0056, 0.0573) | 0.107 | 0.0253 (-0.0120, 0.0627) | 0.183 |
| **36 (n=1365)** | 0.0219 (-0.0050, 0.0489) | 0.111 | 0.0248 (-0.0022, 0.0518) | 0.072 | 0.0068 (-0.0303, 0.0441) | 0.718 |
| **43 (n=1414)** | 0.0252 (0.0035, 0.0470) | 0.023 | 0.0282 (0.0064, 0.0500) | 0.011 | 0.0316 (-0.0022, 0.0655) | 0.067 |
| **53 (n=1429)** | 0.0155 (-0.0034, 0.0345) | 0.109 | 0.0172 (-0.0016, 0.0361) | 0.073 | 0.0132 (-0.2269, 0.0492) | 0.469 |
| **60-64 (n=1507)** | 0.0109 (-0.0069, 0.0287) | 0.231 | 0.0121 (-0.0056, 0.0300) | 0.181 |  |  |
| **MCF, %** | | | | | | |
| **20 (n=1219)** | -0.4085 (-0.6547, -0.1623) | 0.001 | -0.4019 (-0.6537, -0.1501) | 0.002 | 0.0983 (-0.1821, 0.3788) | 0.492 |
| **26 (n=1300)** | -0.6065 (-0.8397, -0.3733) | <0.0001 | -0.5873 (-0.8272, -0.3472) | <0.0001 | -0.0495 (-0.3203, 0.2211) | 0.719 |
| **36 (n=1333)** | -0.4957 (-0.7019, -0.2895) | <0.0001 | -0.4782 (-0.6877, -0.2687) | <0.0001 | 0.2042 (-0.0954, 0.5039) | 0.181 |
| **43 (n=1392)** | -0.5391 (-0.7153, -0.3629) | <0.0001 | -0.5210 (-0.7007, -0.3413) | <0.0001 | 0.1480 (-0.1263, 0.4224) | 0.290 |
| **53 (n=1403)** | -0.5481 (-0.6903, -0.4058) | <0.0001 | -0.5355 (-0.6802, -0.3908) | <0.0001 | 0.0176 (-0.2713, 0.3066) | 0.905 |
| **60-64 (n=1472)** | -0.5930 (-0.7354, -0.4506) | <0.0001 | -0.5909 (-0.7358, -0.4461) | <0.0001 |  |  |

**Model-1: adjusted for age and sex; model-2: adjusted for model-1 plus socioeconomic status/social class and education; and model-3: adjusted for model-2 plus current BMI.**

| **Table S4. Association between WHR at different ages and LV systolic function measured at age 60-64y** | | | | | | |
| --- | --- | --- | --- | --- | --- | --- |
| **Age (y)** | **Model-1** | | **Model-2** | | **Model-3** | |
|  | **Coefficient (95% CI)** | **P value** | **Coefficient (95% CI)** | **P value** | **Coefficient (95% CI)** | **P value** |
| **EF, %** | | | | | | |
| **43y (n=1260)** | -2.187 (-10.11, 5.743) | 0.589 | -3.362 (-11.32, 4.603) | 0.408 | 0.9977 (-7.870, 9.866) | 0.825 |
| **53y (n=1377)** | -8.821 (-15.78, -1.863) | 0.013 | -9.222 (-16.44, -2.003) | 0.012 | -4.297 (-13.08, 4.486) | 0.337 |
| **60-64y (n=1445)** | -10.82 (-17.33, -4.317) | 0.001 | -11.30 (-17.88, -4.721) | 0.001 |  |  |
| **S’, cm/s** | | | | | | |
| **43 (n=1300)** | 0.3492 (-1.332, 2.031) | 0.684 | 0.5631 (-1.165, 2.292) | 0.523 | 0.8530 (-1.049, 2.755) | 0.379 |
| **53 (n=1432)** | 0.3063 (-1.052, 1.664) | 0.658 | 0.5226 (-0.8465, 1.891) | 0.454 | 1.215 (-0.369, 2.801) | 0.133 |
| **60-64 (n=1503)** | -0.666 (-1.929, 0.597) | 0.301 | -0.4747 (-1.751, 0.8025) | 0.466 |  |  |
| **MCF, %** | | | | | | |
| **43 (n=1285)** | -30.52 (-42.77, -18.27) | <0.0001 | -29.74 (-42.33, -17.15) | <0.0001 | -22.98 (-36.88, -9.090) | 0.001 |
| **53 (n=1404)** | -27.37 (-38.21, -16.53) | <0.0001 | -27.37 (-38.34, -16.40) | <0.0001 | -19.16 (-32.96, -5.355) | 0.007 |
| **60-64 (n=1468)** | -24.96 (-34.88, -15.03) | <0.0001 | -24.83 (-34.89, -14.76) | <0.0001 |  |  |

**Model-1: adjusted for age and sex; model-2: adjusted for model-1 plus socioeconomic status/social class and education; and model-3: adjusted for model-2 plus current WHR.**

| **Table S5. Association between BMI at different ages and LV diastolic function measured at age 60-64y** | | | | | | |
| --- | --- | --- | --- | --- | --- | --- |
| **Age (y)** | **Model-1** | | **Model-2** | | **Model-3** | |
|  | **Coefficient (95% CI)** | **P value** | **Coefficient (95% CI)** | **P value** | **Coefficient (95% CI)** | **P value** |
| **E/e’** | | | | | | |
| **20y (n=1285)** | 0.0062 (-0.0386, 0.0510) | 0.786 | -0.0018 (-0.0471, 0.0433) | 0.935 | -0.0850 (-0.1347, -0.0352) | 0.001 |
| **26y (n=1373)** | 0.0600 (0.0198, 0.1001) | 0.003 | 0.0460 (0.0055, 0.0865) | 0.026 | -0.0371 (-0.0853, 0.0111) | 0.132 |
| **36y (n=1415)** | 0.0873 (0.0526, 0.1219) | <0.0001 | 0.0764 (0.0415, 0.1112) | <0.0001 | 0.0028 (-0.0461, 0.0518) | 0.909 |
| **43y (n=1472)** | 0.0916 (0.0626, 0.1207) | <0.0001 | 0.0831 (0.0538, 0.1124) | <0.0001 | 0.0222 (-0.0251, 0.0696) | 0.357 |
| **53y (n=1480)** | 0.0838 (0.0594, 0.1081) | <0.0001 | 0.0786 (0.0541, 0.1031) | <0.0001 | 0.0154 (-0.0347, 0.0656) | 0.546 |
| **60-64y (n=1560)** | 0.0868 (0.0645, 0.1090) | <0.0001 | 0.0806 (0.0582, 0.1030) | <0.0001 |  |  |
| **e’, cm/s** | | | | | | |
| **20y (n=1318)** | -0.0079 (-0.0483, 0.0325) | 0.702 | -0.0012 (-0.0419, 0.0394) | 0.952 | 0.0568 (0.0115, 0.1020) | 0.014 |
| **26y (n=1411)** | -0.0333 (-0.0693, 0.0025) | 0.069 | -0.0226 (-0.0591, 0.0137) | 0.222 | 0.0492 (0.0057, 0.0928) | 0.027 |
| **36y (n=1450)** | -0.0492 (-0.0804, -0.0180) | 0.002 | -0.0418 (-0.0733, -0.0104) | 0.009 | 0.0309 (-0.0134, 0.0754) | 0.172 |
| **43y (n=1509)** | -0.0589 (-0.0853, -0.0325) | <0.0001 | -0.0514 (-0.0780, -0.0248) | <0.0001 | 0.0115 (-0.014, 0.0545) | 0.598 |
| **53y (n=1520)** | -0.0548 (-0.0769, -0.0328) | <0.0001 | -0.0510 (-0.0731, -0.0288) | <0.0001 | 0.0169 (-0.0283, 0.0623) | 0.463 |
| **60-64y (n=1602)** | -0.0658 (-0.0860, -0.0455) | <0.0001 | -0.0606 (-0.0810, -0.0402) | <0.0001 |  |  |
| **LAV indexed to BSA** | | | | | | |
| **20y (n=1162)** | 0.2906 (0.1303, 0.4508) | <0.0001 | 0.2931 (0.1320, 0.4541) | <0.0001 | 0.1228 (-0.0574, 0.3031) | 0.181 |
| **26y (n=1241)** | 0.4663 (0.3001, 0.6324) | <0.0001 | 0.4582 (0.2951, 0.6213) | <0.0001 | 0.3117 (0.1237, 0.4997) | 0.001 |
| **36y (n=1274)** | 0.4881 (0.3530, 0.6232) | <0.0001 | 0.4671 (0.3303, 0.6040) | <0.0001 | 0.3854 (0.1851, 0.5857) | <0.0001 |
| **43y (n=1322)** | 0.3844 (0.2684, 0.5004) | <0.0001 | 0.3669 (0.2489, 0.4849) | <0.0001 | 0.3080 (0.1198, 0.4961) | 0.001 |
| **53y (n=1332)** | 0.2930 (0.1886, 0.3974) | <0.0001 | 0.2855 (0.1808, 0.3903) | <0.0001 | 0.2115 (-0.0004, 0.4235) | 0.050 |
| **60-64y (n=1406)** | 0.2599 (0.1709, 0.3488) | <0.0001 | 0.2462 (0.1553, 0.3372) | <0.0001 |  |  |
| **LAV indexed to height^2^** | | | | | | |
| **20y (n=1170)** | 0.3908 (0.2732, 0.5085) | <0.0001 | 0.3897 (0.2724, 0.5071) | <0.0001 | 0.0890 (-0.0336, 0.2118) | 0.155 |
| **26y (n=1251)** | 0.5285 (0.3962, 0.6607) | <0.0001 | 0.5201 (0.3925, 0.6476) | <0.0001 | 0.2150 (0.0860, 0.3439) | 0.001 |
| **36y (n=1286)** | 0.5722 (0.4751, 0.6693) | <0.0001 | 0.5552 (0.4570, 0.6534) | <0.0001 | 0.2755 (0.1360, 0.4149) | <0.0001 |
| **43y (n=1335)** | 0.4858 (0.4008, 0.5709) | <0.0001 | 0.4720 (0.3856, 0.5584) | <0.0001 | 0.2241 (0.0928, 0.3554) | 0.001 |
| **53y (n=1345)** | 0.4086 (0.3326, 0.4847) | <0.0001 | 0.4021 (0.3257, 0.4785) | <0.0001 | 0.1421 (-0.0059, 0.2903) | 0.060 |
| **60-64y (n=1419)** | 0.4060 (0.3425, 0.4696) | <0.0001 | 0.3962 (0.3312, 0.4612) | <0.0001 |  |  |
| **LAV indexed to height** | | | | | | |
| **20y (n=1170)** | 0.6350 (0.4403, 0.8297) | <0.0001 | 0.6377 (0.4437, 0.83317) | <0.0001 | 0.1461 (-0.0583, 0.3505) | 0.161 |
| **26y (n=1251)** | 0.8640 (0.6489, 1.079) | <0.0001 | 0.8560 (0.6479, 1.064) | <0.0001 | 0.3587 (0.1461, 0.5714) | 0.001 |
| **36y (n=1286)** | 0.9424 (0.7798, 1.104) | <0.0001 | 0.9189 (0.7552, 1.082) | <0.0001 | 0.4796 (0.2476, 0.7117) | <0.0001 |
| **43y (n=1335)** | 0.7851 (0.6428, 0.9274) | <0.0001 | 0.7665 (0.6226, 0.9105) | <0.0001 | 0.3641 (0.1461, 0.5822) | 0.001 |
| **53y (n=1345)** | 0.6666 (0.5407, 0.7925) | <0.0001 | 0.6587 (0.5326, 0.7848) | <0.0001 | 0.2498 (0.0064, 0.4932) | 0.044 |
| **60-64y (n=1419)** | 0.6611 (0.5548, 0.7674) | <0.0001 | 0.6477 (0.5393, 0.7561) | <0.0001 |  |  |

**Model-1: adjusted for age and sex; model-2: adjusted for model-1 plus socioeconomic status/social class and education; and model-3: adjusted for model-2 plus current BMI.**

| **Table S6. Association between WHR at different ages and LV diastolic function measured at age 60-64y** | | | | | | |
| --- | --- | --- | --- | --- | --- | --- |
| **Age (y)** | **Model-1** | | **Model-2** | | **Model-3** | |
|  | **Coefficient (95% CI)** | **P value** | **Coefficient (95% CI)** | **P value** | **Coefficient (95% CI)** | **P value** |
| **E/e’** | | | | | | |
| **43y (n=1360)** | 5.105 (3.211, 6.998) | <0.0001 | 4.377 (2.454, 6.299) | <0.0001 | 2.676 (0.5236, 4.829) | 0.015 |
| **53y (n=1483)** | 4.840 (3.067, 6.614) | <0.0001 | 4.293 (2.504, 6.082) | <0.0001 | 1.788 (-0.4061, 3.982) | 0.110 |
| **60-64y (n=1556)** | 5.308 (3.670, 6.946) | <0.0001 | 4.738 (3.079, 6.398) | <0.0001 |  |  |
| **e’, cm/s** | | | | | | |
| **43y (n=1394)** | -3.732 (-5.438, -2.026) | <0.0001 | -3.197 (-4.933, -1.460) | <0.0001 | -0.5358 (-2.464, 1.392) | 0.586 |
| **53y (n=1523)** | -4.902 (-6.501, -3.304) | <0.0001 | -4.530 (-6.145, -2.914) | <0.0001 | -1.246 (-3.214, 0.7215) | 0.214 |
| **60-64y (n=1598)** | -6.266 (-7.741, -4.792) | <0.0001 | -5.861 (-7.357, -4.366) | <0.0001 |  |  |
| **LAVi to BSA** | | | | | | |
|  |  |  |  |  |  |  |
| **43y (n=1218)** | 11.75 (4.039, 19.46) | 0.003 | 11.07 (3.370, 18.78) | 0.005 | 9.89 (1.474, 18.30) | 0.021 |
| **53y (n=1336)** | 10.40 (3.505, 17.30) | 0.003 | 9.559 (2.577, 16.54) | 0.007 | 8.066 (-0.4407, 16.57) | 0.063 |
| **60-64y (n=1402)** | 9.327 (2.766, 15.88) | 0.005 | 8.101 (1.526, 14.67) | 0.016 |  |  |
| **LAV indexed to height^2^** | | | | | | |
| **43y (n=1231)** | 13.43 (8.065, 18.81) | <0.0001 | 12.71 (7.340, 18.08) | <0.0001 | 9.618 (3.781, 15.45) | 0.001 |
| **53y (n=1349)** | 14.45 (9.571, 19.33) | <0.0001 | 13.63 (8.695, 18.57) | <0.0001 | 10.40 (4.360, 16.44) | 0.001 |
| **60-64y (n=1415)** | 13.04 (8.569, 17.52) | <0.0001 | 11.90 (7.396, 16.40) | <0.0001 |  |  |
| **LAV indexed to height** | | | | | | |
| **43y (n=1231)** | 21.60 (12.50, 30.69) | <0.0001 | 20.71 (11.61, 29.81) | <0.0001 | 15.21 (5.361, 25.07) | 0.003 |
| **53y (n=1349)** | 23.65 (15.45, 31.85) | <0.0001 | 22.57 (14.26, 30.88) | <0.0001 | 16.71 (6.534, 26.89) | 0.001 |
| **60-64y (n=1415)** | 21.97 (14.44, 29.50) | <0.0001 | 20.37 (12.79, 27.95) | <0.0001 |  |  |

**Model-1: adjusted for age and sex; model-2: adjusted for model-1 plus socioeconomic status/social class and education; and model-3: adjusted for model-2 plus current WHR.**

| **Table S7. Association between BMI at different ages and LV structure measured at age 60-64y excluding those with T2DM** | | | | | | |
| --- | --- | --- | --- | --- | --- | --- |
| **Age (y)** | **Model-1** | | **Model-2** | | **Model-3** | |
|  | **Coefficient (95% CI)** | **P value** | **Coefficient (95% CI)** | **P value** | **Coefficient (95% CI)** | **P value** |
| **LV mass, g** | | | | | | |
| **20y (n=1148)** | 3.9756 (2.7571, 5.1940) | <0.0001 | 3.8918 (2.6775, 5.1061) | <0.0001 | 0.2440 (-1.1354, 1.6235) | 0.729 |
| **26y (n=1224)** | 5.4182 (4.0661, 6.7703) | <0.0001 | 5.3063 (3.9752, 6.6375) | <0.0001 | 1.2215 (-0.2520, 2.6950) | 0.104 |
| **36y (n=1254)** | 5.698 (4.7487, 6.6472) | <0.0001 | 5.5987 (4.6515, 6.5460) | <0.0001 | 1.0870 (-0.4704, 2.6445) | 0.171 |
| **43y (n=1310)** | 5.3617 (4.4699, 6.2536) | <0.0001 | 5.2730 (4.3893, 6.1568) | <0.0001 | 1.2708 (-0.1212, 2.6628) | 0.074 |
| **53y (n=1322)** | 5.0080 (4.1897, 5.8264) | <0.0001 | 4.9904 (4.1804, 5.8004) | <0.0001 | 1.9892 (0.1390, 3.8395) | 0.035 |
| **60-64y (n=1385)** | 4.8371 (4.1023, 5.5719) | <0.0001 | 4.7928 (4.0664, 5.5192) | <0.0001 |  |  |
| **LVIDd, cm** | | | | | | |
| **20y (n=1165)** | 0.0361 (0.0233, 0.0490) | <0.0001 | 0.0364 (0.0236, 0.0493) | <0.0001 | 0.0166 (0.0028, 0.0303) | 0.018 |
| **26y (n=1243)** | 0.0437 (0.0309, 0.0564) | <0.0001 | 0.0431 (0.0309, 0.0553) | <0.0001 | 0.0227 (0.0100, 0.0355) | <0.0001 |
| **36y (n=1273)** | 0.0483 (0.0383, 0.0583) | <0.0001 | 0.0468 (0.0367, 0.0569) | <0.0001 | 0.0287 (0.0151, 0.0424) | <0.0001 |
| **43y (n=1331)** | 0.0410 (0.0320, 0.0500) | <0.0001 | 0.0399 (0.0309, 0.0490) | <0.0001 | 0.0266 (0.0134, 0.0397) | <0.0001 |
| **53y (n=1342)** | 0.0321 (0.0245, 0.0396) | <0.0001 | 0.0320 (0.0244, 0.0396) | <0.0001 | 0.0172 (0.002, 0.0328) | 0.030 |
| **60-64y (n=1406)** | 0.0302 (0.0239, 0.0365) | <0.0001 | 0.0293 (0.0229, 0.0358) | <0.0001 |  |  |
| **RWT** | | | | | | |
| **20y (n=1148)** | 0.0014 (-0.0006, 0.0035) | 0.185 | 0.0013 (-0.0007, 0.0034) | 0.216 | -0.0011 (-0.0034, 0.0012) | 0.363 |
| **26y (n=1224)** | 0.0019 (-0.0000, 0.0040) | 0.061 | 0.0018 (-0.0002, 0.0039) | 0.080 | -0.0010 (-0.0034, 0.0014) | 0.418 |
| **36y (n=1254)** | 0.0007 (-0.0010, 0.0025) | 0.416 | 0.0008 (-0.0010, 0.0026) | 0.395 | -0.0038 (-0.0063, -0.0012) | 0.006 |
| **43y (n=1310)** | 0.0018 (0.0003, 0.0033) | 0.022 | 0.0018 (0.0002, 0.0034) | 0.020 | -0.0023 (-0.0047, 0.0006) | 0.057 |
| **53y (n=1322)** | 0.0027 (0.0014, 0.0040) | <0.0001 | 0.0027 (0.0014, 0.0040) | <0.0001 | 0.0002 (-0.0024, 0.0027) | 0.895 |
| **60-64y (n=1385)** | 0.0028 (0.0016, 0.0039) | <0.0001 | 0.0029 (0.0017, 0.0040) | <0.0001 |  |  |

**Model-1: adjusted for age and sex (plus height for LVIDd; and height^1.7^ for LV mass); model-2: adjusted for model-1 plus socioeconomic status/social class and education; and model-3: adjusted for model-2 plus current BMI.**

| **Table S8. Association between BMI at different ages and LV systolic function measured at age 60-64y excluding those with T2DM** | | | | | | |
| --- | --- | --- | --- | --- | --- | --- |
| **Age (y)** | **Model-1** | | **Model-2** | | **Model-3** | |
|  | **Coefficient (95% CI)** | **P value** | **Coefficient (95% CI)** | **P value** | **Coefficient (95% CI)** | **P value** |
| **EF, %** | | | | | | |
| **20y (n=1126)** | -0.0971 (-0.2793, 0.0584) | 0.296 | -0.1043 (-0.2870, 0.0784) | 0.263 | -0.0864 (-0.2841, 0.1111) | 0.391 |
| **26y (n=1204)** | -0.1409 (-0.3053, 0.0233) | 0.093 | -0.1595 (-0.3299, -0.0108) | 0.067 | -0.1474 (-0.3479, 0.0529) | 0.149 |
| **36y (n=1233)** | -0.1191 (-0.2668, 0.0285) | 0.114 | -0.1356 (-0.2890, -0.0177) | 0.083 | -0.1267 (-0.3374, 0.0839) | 0.238 |
| **43y (n=1289)** | -0.0712 (-0.1986, 0.0562) | 0.273 | -0.0795 (-0.2119, 0.0529) | 0.239 | -0.0155 (-0.2156, 0.1845) | 0.879 |
| **53y (n=1293)** | -0.0929 (-0.1994, 0.0135) | 0.087 | -0.0969 (-0.2068, -0.0130) | 0.084 | -0.1156 (-0.3496, 0.1182) | 0.332 |
| **60-64y (n=1362)** | -0.0629 (-0.1560, 0.0302) | 0.185 | -0.0694 (-0.1653, 0.0264) | 0.156 |  |  |
| **S’, cm/s** | | | | | | |
| **20 (n=1160)** | 0.0334 (-0.0020, 0.0689) | 0.065 | 0.0298 (-0.0057, 0.0654) | 0.100 | 0.0194 (-0.0201, 0.0589) | 0.335 |
| **26 (n=1240)** | 0.0267 (-0.0067, 0.0601) | 0.118 | 0.0270 (-0.0067, 0.0609) | 0.116 | 0.0263 (-0.0136, 0.0663) | 0.197 |
| **36 (n=1274)** | 0.0167 (-0.0132, 0.0467) | 0.273 | 0.0190 (-0.0109, 0.0490) | 0.213 | -0.0043 (-0.0452, 0.0365) | 0.835 |
| **43 (n=1321)** | 0.0250 (0.0009, 0.0492) | 0.042 | 0.0277 (0.0035, 0.0520) | 0.025 | 0.0286 (-0.0085, 0.0658) | 0.131 |
| **53 (n=1337)** | 0.0164 (-0.0051, 0.0381) | 0.136 | 0.0177 (-0.0037, 0.0393) | 0.106 | 0.0109 (-0.0297, 0.0515) | 0.598 |
| **60-64 (n=1407)** | 0.0114 (-0.0076, 0.0304) | 0.240 | 0.0121 (-0.0069, 0.0311) | 0.214 |  |  |
| **MCF, %** | | | | | | |
| **20 (n=1146)** | -0.3584 (-0.6248, -0.0920) | 0.008 | -0.3530 (-0.6242, -0.0819) | 0.011 | 0.1194 (-0.1819, 0.4208) | 0.437 |
| **26 (n=1222)** | -0.5425 (-0.7852, -0.2999) | <0.0001 | -0.5314 (-0.7810, -0.2819) | <0.0001 | 0.0005 (-0.2774, 0.2785) | 0.997 |
| **36 (n=1252)** | -0.4669 (-0.6858, -0.2481) | <0.0001 | -0.4605 (-0.6813, -0.2397) | <0.0001 | 0.2594 (-0.0484, 0.5672) | 0.099 |
| **43 (n=1308)** | -0.5144 (-0.7069, -0.3219) | <0.0001 | -0.5039 (-0.6996, -0.3083) | <0.0001 | 0.2117 (-0.0768, 0.5003) | 0.150 |
| **53 (n=1320)** | -0.5768 (-0.7335, -0.4201) | <0.0001 | -0.5746 (-0.7333, -0.4160) | <0.0001 | 0.0391 (-0.3558, 0.2774) | 0.808 |
| **60-64 (n=1383)** | -0.5956 (-0.7442, -0.4470) | <0.0001 | -0.6007 (-0.7518, -0.4495) | <0.0001 |  |  |

**Model-1: adjusted for age and sex; model-2: adjusted for model-1 plus socioeconomic status/social class and education; and model-3: adjusted for model-2 plus current BMI.**

| **Table S9. Association between BMI at different ages and LV diastolic function measured at age 60-64y excluding those with T2DM** | | | | | | |
| --- | --- | --- | --- | --- | --- | --- |
| **Age (y)** | **Model-1** | | **Model-2** | | **Model-3** | |
|  | **Coefficient (95% CI)** | **P value** | **Coefficient (95% CI)** | **P value** | **Coefficient (95% CI)** | **P value** |
| **E/e’** | | | | | | |
| **20y (n=1207)** | -0.0079 (-0.0546, 0.0386) | 0.737 | -0.0113 (-0.0583, 0.0355) | 0.635 | -0.0829 (-0.1347, -0.0311) | 0.002 |
| **26y (n=1288)** | 0.0505 (0.0083, 0.0928) | 0.019 | 0.0398 (-0.0027, 0.0823) | 0.067 | -0.0271 (-0.0772, 0.0229) | 0.288 |
| **36y (n=1327)** | 0.0815 (0.0439, 0.1190) | <0.0001 | 0.0720 (0.0343, 0.1098) | <0.0001 | 0.0159 (-0.0368, 0.0686) | 0.554 |
| **43y (n=1382)** | 0.0800 (0.0485, 0.1114) | <0.0001 | 0.0740 (0.0424, 0.1056) | <0.0001 | 0.0231 (-0.0268, 0.0731) | 0.363 |
| **53y (n=1393)** | 0.0753 (0.0486, 0.1020) | <0.0001 | 0.0706 (0.0439, 0.0974) | <0.0001 | 0.0157 (-0.0379, 0.0694) | 0.565 |
| **60-64y (n=1465)** | 0.0767 (0.0531, 0.1003) | <0.0001 | 0.0714 (0.0477, 0.0951) | <0.0001 |  |  |
| **e’, cm/s** | | | | | | |
| **20y (n=1235)** | -0.0005 (-0.0440, 0.0428) | 0.979 | 0.0041 (-0.0395, 0.0478) | 0.853 | 0.0678 (0.0195, 0.1160) | 0.006 |
| **26y (n=1322)** | -0.0368 (-0.0758, 0.0020) | 0.063 | -0.0274 (-0.0668, 0.0119) | 0.172 | 0.0451 (-0.0011, 0.0914) | 0.056 |
| **36y (n=1358)** | -0.0577 (-0.0926, -0.0228) | 0.001 | -0.0509 (-0.0860, -0.0158) | 0.005 | 0.0266 (-0.0223, 0.0757) | 0.286 |
| **43y (n=1415)** | -0.0645 (-0.0940, -0.0350) | <0.0001 | -0.0585 (-0.0881, -0.0288) | <0.0001 | 0.006 (-0.0361, 0.0574) | 0.655 |
| **53y (n=1427)** | -0.0663 (-0.0912, -0.0413) | <0.0001 | -0.0628 (-0.0878, -0.0377) | <0.0001 | 0.0051 (-0.0447, 0.0550) | 0.840 |
| **60-64y (n=1501)** | -0.0734 (-0.0955, -0.0513) | <0.0001 | -0.0684 (-0.0907, -0.0461) | <0.0001 |  |  |
| **LAVi to BSA** | | | | | | |
| **20y (n=1091)** | 0.2887 (0.1194, 0.4581) | 0.001 | 0.3007 (0.1301, 0.4713) | 0.001 | 0.1457 (-0.0441, 0.3357) | 0.132 |
| **26y (n=1164)** | 0.4559 (0.2758, 0.6360) | <0.0001 | 0.4541 (0.2770, 0.6312) | <0.0001 | 0.3094 (0.1117, 0.5070) | 0.002 |
| **36y (n=1194)** | 0.5097 (0.3620, 0.6574) | <0.0001 | 0.4976 (0.3480, 0.6471) | <0.0001 | 0.4468 (0.2276, 0.6661) | <0.0001 |
| **43y (n=1240)** | 0.4176 (0.2947, 0.5406) | <0.0001 | 0.4037 (0.2785, 0.5288) | <0.0001 | 0.3958 (0.1964, 0.5952) | <0.0001 |
| **53y (n=1250)** | 0.3010 (0.1862, 0.4157) | <0.0001 | 0.3027 (0.1872, 0.4182) | <0.0001 | 0.2790 (0.0421, 0.5158) | 0.021 |
| **60-64y (n=1317)** | 0.2492 (0.1543, 0.3442) | <0.0001 | 0.2424 (0.1443, 0.3405) | <0.0001 |  |  |

**Model-1: adjusted for age and sex; model-2: adjusted for model-1 plus socioeconomic status/social class and education; and model-3: adjusted for model-2 plus current BMI.**

**Figure-S5. Association between BMI at different ages and LV structure measured at age 60-64y in men vs in women.** A) LV mass, B) LVIDd, C) RWT. Model-1 (top): adjusted for age and sex (plus height for LVIDd; and height^1.7^ for LV mass); model-2 (middle): adjusted for model-1 plus socioeconomic status/social class and education; and model-3 (bottom): adjusted for model-2 plus current BMI.

**Figure-S6. Association between BMI at different ages and LV diastolic function measured at age 60-64y in men vs in women.** A) E/e’, B) e’, C) LAVi to BSA. Model-1 (top): adjusted for age and sex; model-2 (middle): adjusted for model-1 plus socioeconomic status/social class and education; and model-3 (bottom): adjusted for model-2 plus current BMI.

**Figure-S7. Association between BMI at different ages and LV systolic function measured at age 60-64y in men vs in women.** A) EF, B) S’, C) MCF. Model-1 (top): adjusted for age and sex; model-2 (middle): adjusted for model-1 plus socioeconomic status/social class and education; and model-3 (bottom): adjusted for model-2 plus current BMI.
